# A Patch-Aligned Multimodal Deep Learning Framework for Non-Destructive Freshness Monitoring of Postharvest Mushrooms Using Hyperspectral Imaging

**DOI:** 10.3390/foods15183224

**Published:** 2026-09-11

**Authors:** Zhen Guo, Yaru Wang, Lele Cao, Fernando A. Auat-Cheein, Xingfeng Guo

**Affiliations:** 1 Shandong Key Laboratory of Applied Technology for Protein and Peptide Drugs, School of Pharmaceutical Sciences and Food Engineering, Liaocheng University, Liaocheng 252000, China; 13176270429@163.com (Y.W.); caolele@lcu.edu.cn (L.C.); 2 Department of Electronic Engineering, Universidad Técnica Federico Santa María, Av. España 1680, Valparaiso 2390123, Chile; fauat@harper-adams.ac.uk; 3 Department of Agricultural Engineering, Harper Adams University, Newport TF10 8NB, UK

**Keywords:** multimodal deep learning, non-destructive testing, Vis-NIR hyperspectral imaging, food quality assessment, cold-chain logistics, *Agaricus bisporus*

## Abstract

Button mushrooms (*Agaricus bisporus*) are highly perishable, making rapid and automated postharvest freshness evaluation crucial for cold-chain logistics. Visible-near-infrared (Vis-NIR) hyperspectral imaging is promising for non-destructive food quality assessment, yet mining highly redundant spatial–spectral data remains challenging. This study proposes a novel artificial intelligence approach, the patch-aligned multimodal interaction fusion network (PAMIF-Net), to monitor postharvest mushroom freshness. Using a Vis-NIR dataset of 400 *A. bisporus* caps over a 9-day refrigerated storage period, this deep learning architecture dynamically fuses global spectral features (indicating internal physicochemical shifts) with localized spatial morphological features (capturing surface deterioration) using a gated attention mechanism. Extensive evaluations across 10 independent trials demonstrated that PAMIF-Net achieved optimal classification accuracy (up to 100% on the current test set) and a minimal mean absolute error for five-class storage time recognition. Furthermore, it exhibited superior computational efficiency and significantly lower inference latency compared to classical machine learning and standard deep learning backbones. This multimodal spatial–spectral deep learning framework demonstrates the feasibility of combining HSI and deep learning for the specific task of automated storage time recognition of *A. bisporus* under controlled refrigerated conditions.

## 1. Introduction

Button mushrooms (*Agaricus bisporus*, *A. bisporus*) are widely consumed as a common edible fungus and is regarded as an important source of nutrition and bioactive compounds [1,2]. It is characterized by high protein content and is also rich in amino acids, vitamins, minerals, polysaccharides, phenolic compounds, and ergosterol [3,4]. The potential health benefits of edible mushrooms are increasingly emphasized, and their intake is associated with the prevention or management of chronic diseases such as cancer, obesity, and hypertension [5]. At the same time, button mushroom is favored in plant-based diets and is accepted as a low-fat, low-calorie alternative to meat [6,7]. However, *A. bisporus* is also recognized as a highly perishable commodity, because of its high moisture content, high respiration rate, and absence of a protective cuticle barrier [8,9]. After harvest, rapid quality deterioration is typically observed during storage, and this deterioration is driven by enzymatic browning, texture softening, water loss, and microbial growth [10,11]. As a result, the commercial shelf-life of fresh button mushrooms is very limited. Reported values indicate a storage life of only 1–3 days at 20–25 °C and 5–7 days under refrigeration at 0–2 °C [11,12]. Similar observations are reported for other studies, in which *A. bisporus* is described as having a shelf-life of less than 3 days at room temperature and less than about 8–10 days under cold storage [13]. This rapid loss of visual quality, firmness, and sensory acceptance is directly linked to loss of market value and reduced consumer willingness to purchase, and it is also linked to possible food safety concerns when aging mushrooms with visible browning or slimy texture are re-labeled and sold as fresh product [14,15]. For this reason, the reliable tracking of storage duration and optical deterioration along the postharvest chain is regarded as an essential step toward better supply-chain management.

Quality assessment in the mushroom industry is still mainly based on sensory inspection, microbiological evaluation, enzymatic assays, and measurements of color and texture [16,17]. These conventional methods are often destructive, time-consuming, and laboratory-dependent, and their performance is limited for real-time decision-making during distribution and retail [18]. Emerging approaches such as smart labels and pH indicators are proposed for on-package monitoring, but concerns remain over chemical migration, cost, temperature sensitivity, and practical deployment in large-scale food chains [19,20]. In this context, rapid and non-destructive inspection is regarded as a necessary direction for fresh mushroom control. Hyperspectral imaging is presented as a promising alternative because spatial information and spectral information are acquired simultaneously, and both chemical composition and physical structure can be analyzed without contact or sample preparation [21,22]. Hyperspectral imaging has already been applied to postharvest products, including mushrooms, for tasks such as browning assessment, texture evaluation, water content estimation, and detection of mechanical damage or microbial spoilage [21,22,23,24]. Therefore, hyperspectral imaging is considered a suitable foundation for objective, fast, and non-destructive estimation of remaining shelf-life and freshness status in *A. bisporus*, and it is viewed as a feasible tool for preventing economic loss and reducing safety risks in the mushroom supply chain.

Hyperspectral imaging is characterized by very high spectral resolution, and each pixel contains a full reflectance curve across hundreds of contiguous wavelengths, so the hyperspectral cube simultaneously preserves spatial structure and spectral composition [25]. As a result, HSI data are inherently high dimensional, highly redundant, and often sensitive to noise. Large portions of neighboring bands carry overlapping chemical information, and this redundancy limits direct modeling because it increases computational burden and promotes overfitting while not necessarily improving discrimination [26]. At the same time, postharvest mushrooms present complex and spatially non-uniform deterioration patterns such as localized browning, moisture loss, and textural collapse, which means that relevant quality cues are not only spectral but also spatial and structural. It is therefore recognized that the spectrum of a single pixel is not always sufficient, because two different regions may show similar mean spectra but differ in spatial texture, morphology, or local interaction patterns. For this reason, joint use of spectral information and spatial information is considered necessary to achieve reliable freshness assessment.

To address these issues, multi-dimensional feature extraction has been proposed. In this strategy, the hyperspectral cube is not treated as a single averaged spectrum, but is decomposed into complementary descriptors that reflect different physical aspects of the sample. Low-level spectral descriptors are used to summarize global chemical trends such as water-related absorption [27], while color and texture descriptors are used to describe visually interpretable surface changes such as browning and cap roughening [28]. Learned spatial–spectral embeddings are used to capture nonlinear correlations and interactions among bands and among local regions of contamination classifying in peanut kernels [26]. Such features can then be fused in a structured manner. Classical fusion approaches are described at the measurement level, the feature level, and the decision level [29]. These approaches aim to merge information from different sources while preserving both local statistics and global structure. Recent work further emphasizes that an effective fusion strategy should maintain the spatial–spectral relationship, avoid the loss of localized deterioration cues, and at the same time remain interpretable and traceable to physical properties, rather than behaving as an opaque black box.

This study was conducted to address the problem of rapid quality deterioration of button mushrooms during storage and the lack of an objective, rapid, and non-destructive quantitative evaluation method. Therefore, the overall objective of this study was to develop a non-destructive, multimodal deep learning framework (PAMIF-Net) based on Vis-NIR hyperspectral imaging for evaluating the storage time of postharvest button mushrooms. To achieve this, three specific objectives were established: (1) to characterize the spatial and spectral evolution of mushroom caps associated with progressive deterioration (browning, moisture loss) during refrigerated storage; (2) to extract and synergistically fuse complementary multi-dimensional features, including global spectra, color–texture, local patch states, and spatial–spectral embeddings, using a novel patch-aligned architecture; and (3) to evaluate the classification accuracy, stability, and computational efficiency of the proposed network against traditional machine learning and standard deep learning models.

## 2. Materials and Methods

### 2.1. Sample Preparation

The common edible mushroom *A. bisporus* was selected as the research subject to investigate quality variation during storage and develop a non-destructive freshness recognition strategy. Fresh *A. bisporus* samples were purchased from two different local supermarkets in Dongchangfu District, Liaocheng, China, in two independent harvest batches. A total of 400 mushroom caps with diameters of 4 ± 0.5 cm, free of visible mechanical damage or disease lesions, were screened for the formal experiment. The stipes of all selected mushrooms were removed, and the caps were labelled with unique sample identifiers. All samples were pre-cooled overnight at 4 °C with a relative humidity (RH) of 80%, and then stored under constant refrigerated conditions in the laboratory (4 ± 1 °C, RH 80 ± 5%).

A parallel sampling design was adopted in this study, rather than continuous tracking of individual mushroom samples throughout the entire storage period. Specifically, 80 independent parallel samples were randomly selected for hyperspectral image acquisition at each preset sampling time point (day 1, day 3, day 5, day 7, and day 9 of storage). It is important to clarify that chronological storage time was utilized in this study as a practical proxy for mushroom freshness. Although independent physicochemical, microbiological, or sensory reference variables were not recorded to directly quantify internal freshness, existing postharvest literature confirms that under strictly controlled refrigerated conditions (4 ± 1 °C, RH 80 ± 5%), the chronological aging of *A. bisporus* highly correlates with predictable physiological deterioration, such as enzymatic browning and moisture loss. Therefore, classifying the chronological storage duration serves as an objective baseline for modeling the temporal evolution of mushroom quality. Each hyperspectral image contained 10 mushrooms arranged in 5 rows and 2 columns, and 8 hyperspectral images were acquired for all qualified samples per sampling day. The 400 total samples were evenly allocated to the two independent harvest batches, with 200 samples per batch and 40 samples from each batch assigned to each of the 5 sampling time points. The two independent harvest batches were obtained from separate production bases, and were subjected to independent transportation, pre-cooling, storage, and hyperspectral image acquisition procedures. No cross-contamination of sample handling was performed between the two batches throughout the experiment. This independent batch design was adopted to introduce natural batch-to-batch variation, serving as a basis to test the algorithm’s stability across a short demonstration dataset. It should be noted that for the formal model evaluation, the samples from both batches were combined, and a repeated stratified hold-out testing protocol was employed to evaluate the algorithm, rather than a strict cross-batch (train on Batch 1, test on Batch 2) validation.

### 2.2. Hyperspectral Images Acquisition and Processing

**Figure 1:**
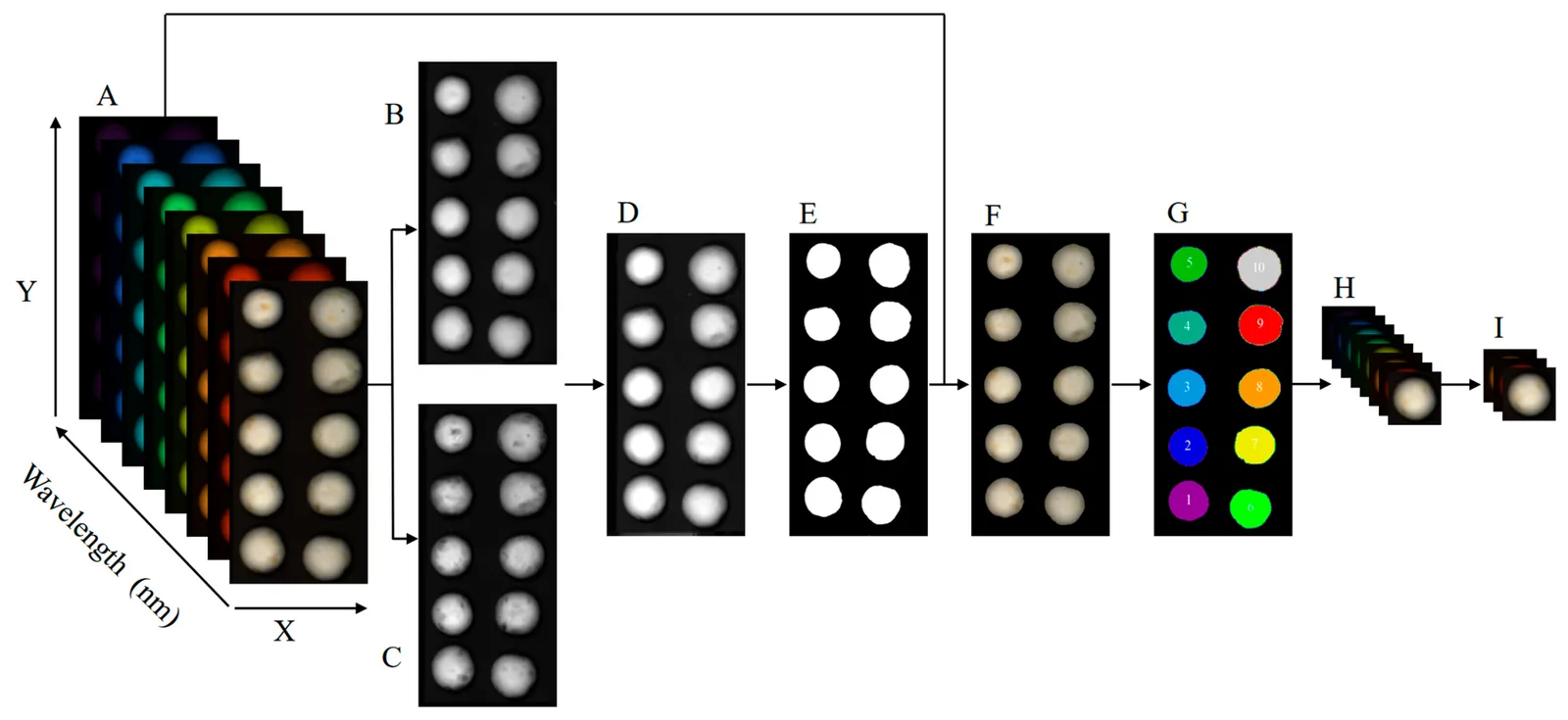
Hyperspectral images processing and threshold segmentation: (**A**) raw hyperspectral cube, (**B**) gray image of 509.3 nm, (**C**) gray image of 841.5 nm, (**D**) band-ratio image, (**E**) binary mask image, (**F**) background-removed hyperspectral image, (**G**) labeled image, (**H**) hyperspectral image of individual mushroom, and (**I**) individual hyperspectral image after PCA.

A visible–near-infrared hyperspectral imaging system (Isuzu Optics Corp., Taiwan, China) was used to acquire hyperspectral images from mushrooms. The spectral range was 400–1000 nm with a spectral resolution of approximately 2.4 nm, providing 226 bands in total. To ensure imaging quality, a stable and controllable artificial light source was employed. Standardized preprocessing included dark-current and white-reference correction, spatial registration, and geometric cropping. To eliminate orientation variability and standardize the machine learning inputs, all 400 sample caps in the formal experiment were consistently positioned and imaged facing upward (front cap cuticle exposed to the sensor). While exploratory spectral scans of the back (gill side) were conducted to illustrate anatomical differences (as discussed in Section 3.1), only the uniform front-facing cap regions were defined as the analysis area (ROI) for the classification dataset, as shown in Figure 1. Grayscale images at 509.3 nm (Figure 1B) and 841.5 nm (Figure 1C) were ratioed to form a band-ratio image (Figure 1D). These specific wavelengths were empirically selected because 509.3 nm (in the visible region) exhibited strong relative absorption by the mushroom cap, whereas 841.5 nm (in the near-infrared region) showed high reflectance due to internal tissue scattering. The ratio of these two bands maximized the contrast between the mushroom ROI and the dark background, facilitating robust bimodal segmentation using Otsu’s method. The resulting binary mask (Figure 1E) was multiplied with the original hyperspectral cube on a band-by-band basis to remove background (Figure 1F). Principal component analysis (PCA) was then performed, and the first 10 principal components were retained (Figure 1H). For each mushroom, the minimum bounding rectangle was computed to obtain the cap’s pixel length and width, and a 150 × 150-pixel patch was cropped as the standard ROI for subsequent analysis (Figure 1I).

### 2.3. Multi-Dimensional Features Extraction

**Figure 2:**
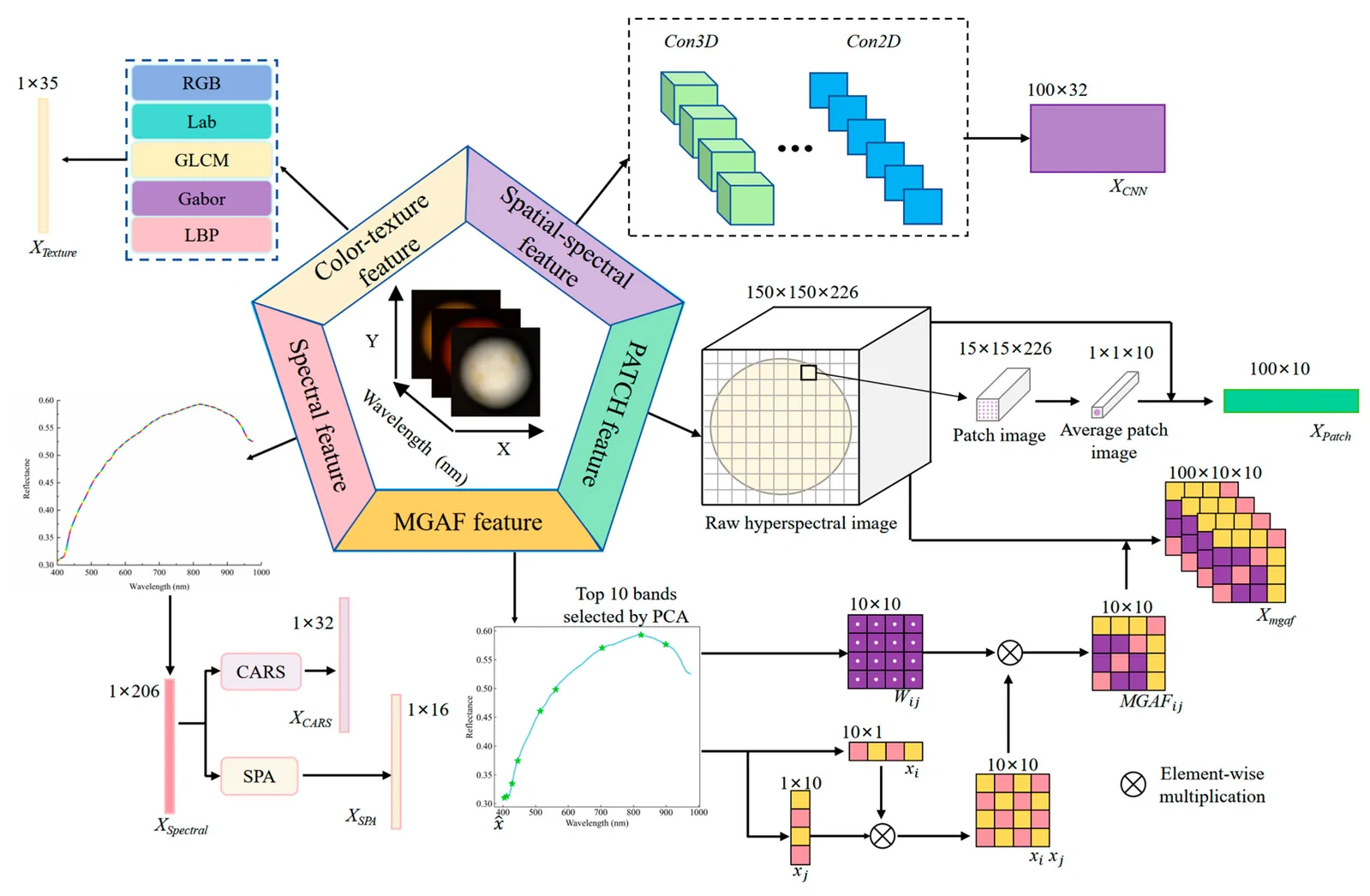
Process of multi-dimensional features extraction.

To evaluate the storage time of mushrooms from a single, masked hyperspectral ROI without direct biochemical sampling, a multi-dimensional feature space was designed to fuse complementary cues across spectral, colorimetric, textural, meso-scale, and learned spectral–spatial levels, as depicted in Figure 2. After background removal and PCA preprocessing (Section 2.2), each ROI was represented by 5 coordinated descriptors with matched geometry: (i) a compact color–texture vector summarizing human-perceivable appearance, (ii) a global spectral vector capturing the full reflectance profile, (iii) a CNN token matrix learning higher-order spectral–spatial features. Together, these descriptors formed a structured representation, (iv) a patch token matrix preserving local spectral states over a fixed spatial grid, and (v) an MGAF tensor encoding within-patch inter-component interactions. Together, these descriptors formed a structured representation {*X_texture_*, *X_spectral_*, *X_cnn_*, *X_patch_*, *X_mgaf_*} that was aligned with the physical 10 × 10 patch layout and was ready for downstream modeling.

This design was motivated by the observation that storage-related deterioration manifested concurrently as chromatic drift, micro-texture evolution, and localized spectral anomalies. Single-family features were unlikely to capture all pathways; therefore, information was deliberately diversified but organized. PCA was first applied to orthogonalize bands and stabilize subsequent statistics, providing a common low-dimensional spectral basis that propagated consistently into the patch, MGAF, and CNN branches (Figure 2). The global spectral vector summarized broad chemical/physical trends; the color/Lab–GLCM–Gabor–LBP vector offered interpretable appearance and microstructure cues that were robust to moderate illumination drift; the patch tokens retained meso-scale heterogeneity by describing each 15 × 15 region with a 10-D spectral embedding; the MGAF tensor introduced an interaction view by weighting pairwise relations between PCA components with a proximity kernel, privileging spectrally adjacent couplings; and the lightweight 3D-CNN → 2D-CNN distilled hierarchical spectral–spatial context into 100 tokens while preserving grid topology.

Two methodological innovations were emphasized. First, patch-wise organization was used as the backbone for all learned and hand-crafted branches, ensuring that every descriptor could be registered and fused at the same 10 × 10 resolution. This patch alignment improved interpretability, supported localized quality assessment, and reduced sensitivity to non-uniform aging. Second, an MGAF representation was introduced to complement patch spectra with an explicit interaction field among PCA components. By emphasizing neighborhood relations in component index space, the MGAF tensor captured coordinated shifts that were diagnostically relevant but not fully expressed by spectra alone. In parallel, the compact 3D-CNN–2D-CNN block leveraged continuity along wavelength and space to learn subtle multi-band motifs with low computational cost, yielding a 100 × 32 token matrix that remained compatible with the patch grid and readily fused with other descriptors. The resulting multi-dimensional feature set was significant for freshness evaluation because it integrated evidence across scales—global appearance, micro-texture, local spectral states, intra-spectral interactions, and hierarchical spectral–spatial patterns—while remaining physically interpretable and model-agnostic. By design, each component addressed a distinct failure mode, and their concatenation provided a robust, information-rich representation of cap condition over time.

#### 2.3.1. Color Features and Texture Features Extraction

Global color descriptors summarized appearance changes that human inspectors also perceive. Three quasi-visible bands were used to form a pseudo-RGB rendering, simple distributional moments were also computed over the cap ROI, including means, variances, skewness, kurtosis, and histogram entropy. Means and variances tracked gradual darkening and contrast loss, skewness and kurtosis captured asymmetric tail behavior caused by spotty browning or localized glare, entropy reflected the spread of tonal states as tissues aged. Additionally, mapping the normalized ROI to CIE-Lab added a device-independent, perceptually quasi-uniform space where L separated lightness from a and b chroma. This made the features more robust to moderate illumination drift and better aligned them with biochemical color changes, providing physically meaningful signals with minimal model complexity.

Texture descriptors targeted meso- and micro-scale organization of the cap surface that color alone could not explain. Gray-level co-occurrence matrices (GLCM) [30] captured second-order spatial statistics, so that contrast, energy, homogeneity, and correlation responded to pore opening, cuticle roughening, and bruise halos that developed during storage. Gabor filters [31] offered localized, orientation-selective band-pass responses; their means and dispersions summarized anisotropy and dominant spatial frequencies linked to micro-wrinkling and fiber alignment. Local binary patterns [32] encoded microstructures via invariant thresholding and were relatively stable under monotonic lighting changes, which was advantageous for day-to-day imaging. Together, these descriptors quantified progression from smooth, taut surfaces to rough, heterogeneous ones, complementing the global color trends.

#### 2.3.2. Spatial–Spectral Feature Extraction

**Figure 3:**
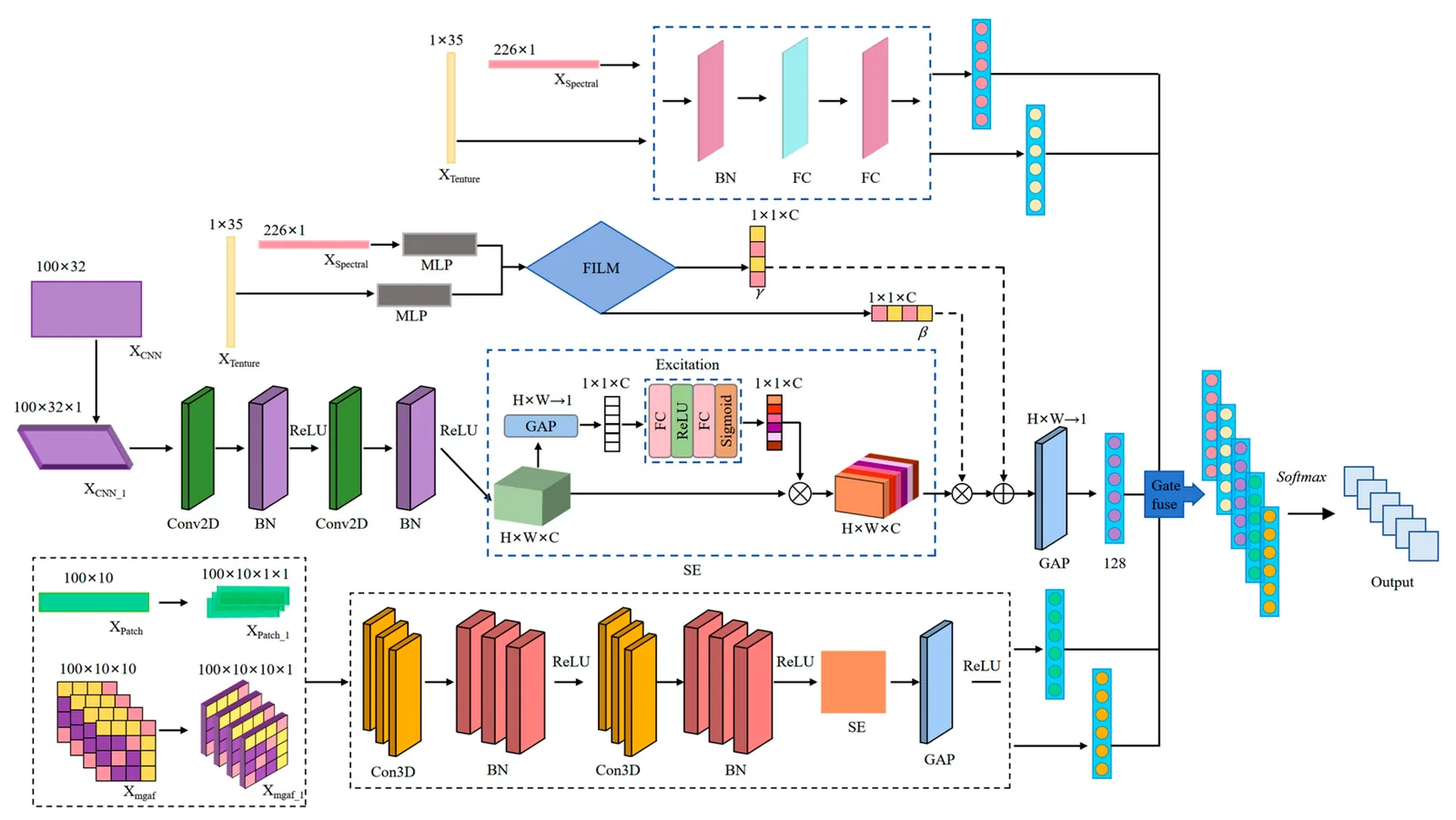
Architecture of the PAMIF-Net.

To learn joint spatial–spectral patterns from a single mushroom ROI, the PCA-reduced cube $X_{PCA}\in R^{150\times 150\times 10}$ (10 principal components) was fed to a lightweight 3D-CNN-2D-CNN. This pipeline matched the implementation in Figure 3 (Conv3D → MaxPool3D → Conv3D → MaxPool3D → reshape → Conv2D → Conv2D → reshape) and the target output shape (100 × 32). All convolutions used “same” padding. Convolutions across space and spectrum were first performed with a 3D layer (32 kernels, 9 × 9 × 3) followed by 3D max-pooling (3 × 3 × 5), thereby aggregating local context while down-sampling both spatial axes and the spectral axis. A second 3D convolution (64 kernels, 5 × 5 × 1) and 3D max-pooling (5 × 5 × 2) further condensed the volume. The pooled tensor was then reshaped from (*H*, *W*, *S*, *C*) = (10, 10, 1, 64) to (10, 10, 64), after which two 2D convolutions (64 kernels, 5 × 5; then 32 kernels, 3 × 3) were applied to enhance spatial discrimination within each spectral component. Finally, the feature map was unfolded on a 10 × 10 spatial grid to produce $X_{CNN}\in R^{100\times 32}$: 100 patch tokens with a 32-dimensional embedding per token.

This design offered three advantages. (i) 3D convolutions in the entrance block exploited smooth correlation along wavelength together with local spatial structure, thereby capturing coupled color/scattering signatures associated with browning and moisture loss. (ii) Pooling along λ and (*x*, *y*) suppressed high-frequency sensor noise and minor misalignments while retaining salient meso-scale patterns. (iii) The final 100 × 32 representation provided a compact, order-preserving tokenization of the cap surface that aligned with the physical 10 × 10 patch layout used elsewhere in the pipeline. Overall, the CNN stage transformed each hyperspectral ROI into a robust set of spatial–spectral embeddings that were well suited for freshness discrimination at the single-mushroom level.

#### 2.3.3. Patch Feature

For each labeled mushroom, the ROI (150 × 150 pixels, *B* bands) was partitioned into a fixed 10 × 10 grid of non-overlapping patches (each 15 × 15 pixels) to preserve meso-scale heterogeneity (Figure 2). Within patch *n* the mean spectral vector $s_{n}\in R^{B}$ was computed to stabilize local spectra against pixel noise,

(1) $$s_{n}=\frac{1}{\left|\Omega_{n}\right|}\sum_{p\in \Omega_{n}}x\left(p\right)$$

where *x*(*p*) denoted the *B*-band spectrum at pixel *p* and $\Omega_{n}$ was the patch support. A global PCA, fitted on the training cubes to decorrelate bands and concentrate variance related to storage-induced changes, was then used to project each *s_n_* onto the first *K* = 10 principal directions:

(2) $$z_{n}=W^{T}\left(s_{n}-\bar{s}\right)\in R^{10}$$

with loading matrix $W\in R^{B\times 10}$ and mean spectrum $\bar{s}$. Stacking ${\left\{z_{n}\right\}}_{n=1}^{100}$ in scanline order yielded a structured tensor:

(3) $$X_{patch}\in R^{100\times 10}$$

where 100 spatial tokens (one per patch) each carrying a compact 10-D spectral embedding. This PCA-guided, patch-wise representation retained local biochemical/physical signatures while significantly reducing redundancy and improving comparability across mushrooms through a common spectral basis.

#### 2.3.4. MGAF Feature

To explicitly encode inter-band relationships within each patch, a PCA-guided multi-granularity gramian angular field (MGAF) representation was constructed from *z_n_*. First, *z_n_* was standardized to ${\hat{z}}_{n}=\left(z_{n}-\mu \right)∕\sigma$. A Gaussian proximity kernel $W_{\sigma }\in R^{K\times K}$ with entries

(4) $${\left(W_{\sigma }\right)}_{ij}=exp\left(-\frac{{\left(i-j\right)}^{2}}{2\sigma^{2}}\right),\;\sigma =2$$

was applied to emphasize interactions between spectrally adjacent components. The MGAF matrix for patch *n* was then defined as

(5) $$M_{n}^{\left(mgaf\right)}=W_{\sigma }⨀\sqrt{{\hat{z}}_{n}{\hat{z}}_{n}^{T}{-\left(1-{{\hat{z}}_{n}^{\circ }}^{2}\right)\left(1-{{\hat{z}}_{n}^{\circ }}^{2}\right)}^{T}}$$

where ∘ denoted element-wise power and ⨀ the Hadamard product. Each $M_{n}^{\left(mgaf\right)}$ captured nonlinear pairwise couplings among the 10 PCA components while privileging local spectral neighborhoods. Collecting all patches produced a third-order tensor:

(6) $$X_{mgaf}\in R^{100\times 10\times 10}$$

which complemented *X_patch_* by representing correlation structure rather than only the reduced spectra. The combined use of *X_patch_* (local spectral states) and *X_mgaf_* (local spectral interactions) provided a structurally separated and information-rich description of spatially localized optical cues on mushroom caps.

### 2.4. Model Construction of PAMIF-Net

A patch-aligned multimodal interaction fusion network (PAMIF-Net) was designed to integrate 5 complementary descriptors extracted from each single-mushroom ROI (Figure 3): a spatial–spectral token map $X_{CNN}\in R^{100\times 32}$, a patch tensor $X_{PATCH}\in R^{100\times 10}$, an interaction tensor $X_{MGAF}\in R^{100\times 10\times 10}$, a global spectrum $X_{Spectral}\in R^{226\times 1}$, and a color–texture vector $X_{Texture}\in R^{35\times 1}$. Each branch transformed its input into a 128-dimensional modality token, after which a light gating unit produced a fused representation for five-class storage time recognition. The computational graph followed the scheme shown in Figure 3, comprising convolutional feature extractors, feature-wise linear modulation, squeeze–excitation re-weighting, and a soft gated fusion head. Shapes evolved along each path as follows: (i) *X_CNN_*: (100 × 32) → *X_CNN_1_*: (100 × 32 × 1) → Conv2D/SE → *FiLM* → *GAP* → 32; (ii) *X_PATCH_*: (100 × 10) → *X_PATCH_1_*: (100 × 10 × 1 × 1) → Conv3D/SE → *FiLM* → *GAP* → 32; (iii) *X_MGAF_*: (100 × 10 × 10) → *X_PATCH_1_*: (100 × 10 × 10 × 1) → Conv3D/SE → *FiLM* → *GAP* → 32; (iv) *X_Spectral_*: (226 × 1) → BN → FC(128) → FC(32) → 32; (v) *X_Texture_*: (35 × 1) → BN → FC(128) → FC(32) → 32; (vi) Tokens (T × 32) → gated fusion $z\in R^{32}$ → five logits. These transformations matched the training pipeline and ensured that all modalities were co-registered on the same 10 × 10 topology (or the unfolded set of 100 tokens) before fusion.

In the CNN pathway (spatial–spectral tokens with conditional modulation), when arranged on the 10 × 10 grid, *X_CNN_* was processed by 2 convolution–normalization–activation blocks to enhance spatial discrimination while preserving the order of the 100 tokens. Channel attention was then applied with a squeeze–excitation (SE) operator:

(7) $$s=\sigma (W_{2}\delta (W_{1}GAP\left(F\right))),\;\;\tilde{F}=F⊙reshapes(s)$$

where *GAP* denoted global average pooling, *δ* is ReLU, and *σ* is the logistic function. To inject global priors carried by spectra and color, feature-wise linear modulation (*FiLM*) was applied to the feature map before pooling. A conditioning vector $c\in R^{32}$, obtained from the spectral and/or color streams (see below), generated channel-wise scale and shift,

(8) $$\gamma =\tanh\left(W_{\gamma }c\right),\;\;\beta =\tanh\left(W_{\beta }c\right),\;\;FiLM\left(F\left|\gamma ,\beta \right.\right)=\gamma ⨀F+\beta$$

with *γ*, *β* reshaped to the channel dimension. After *FiLM*, global pooling yielded a token that was projected to $R^{32}$. This path aligned learned spatial features with biochemical trends indicated by the conditioning streams.

In the patch and mgaf pathways (meso-scale evidence). Both *X_PATCH_* and *X_MGAF_* were treated as light volumetric inputs whose spatial axes followed the fixed 10 × 10 grid and whose depth enumerated patch positions (plus an extra 10-channel interaction dimension for MGAF). Each pathway comprised two 3-D convolution–normalization–activation stages, an SE re-weighting, and global average pooling over (H,W,D) to produce a token in $R^{32}$. The patch pathway summarized local spectral states per grid cell, whereas the MGAF pathway summarized within-patch inter-component relations captured by the affinity field (Section 2.3.4), thus providing complementary information on localized deterioration patterns.

In the spectral and color–texture pathways (tabular towers and conditioners), the full reflectance profile *X_Spectral_* was batch-normalized and projected through two fully connected layers (128 → 32) to form a token ${t_{spec}\in R}^{32}$. The hand-crafted appearance vector *X_Texture_* was normalized and mapped by a compact multilayer perceptron to ${t_{color}\in R}^{32}$. These tokens served a dual role: (i) they entered the fusion head as independent evidence and (ii) they acted as the conditioning source c for *FiLM* in the CNN pathway, enabling sample-wise, top-down modulation from interpretable priors.

Finally, in the gated multimodal fusion and classifier, let ${{\left\{t_{m}\right\}}_{m=1}^{T}\in R}^{32}$ denoted the tokens produced by the active branches (CNN, Patch, MGAF, Spectral and Color). A lightweight gate computed per-modality importances and produced a compact shared descriptor,

(9) $${T=\left[t_{1},\ldots ,t_{T}\right]\in R}^{T\times 32},\;\;w=softmax\left(Ta\right)\in R^{T},\;\;z=\sum_{m=1}^{T}w_{m}t_{m}\in R^{32}$$

where $a\in R^{32}$ was a learned vector. A final linear head produced five logits corresponding to the ordinal storage times (days 1, 3, 5, 7, and 9), which represented the progressive stages of storage-induced deterioration. This head avoided heavy cross-modal attention while retaining adaptability to missing or weak modalities.

PAMIF-Net addressed three design needs specific to freshness assessment. First, patch-aligned multimodality ensured that all descriptors—learned or hand-crafted—were expressed on a common grid, enabling interpretable, location-aware fusion and robustness to spatially non-uniform aging. Second, conditional modulation via *FiLM* allowed global spectral/color evidence to re-weight CNN channels before pooling, encouraging the network to emphasize spectral–textural motifs consistent with biochemical processes such as browning and moisture loss. Third, a soft gated fusion head learned per-sample modality importances with minimal parameters, yielding a compact shared descriptor suitable for small-to-medium datasets. Altogether, PAMIF-Net provided a principled and scalable way to combine global appearance, microtexture, local spectral states, intra-spectral interactions, and hierarchical spatial–spectral patterns into a single, robust representation for single-mushroom freshness recognition.

### 2.5. Other Comparison Methods

In addition to the proposed patch-aligned multimodal interaction fusion network, several conventional methods were implemented for comparison. First, representative deep learning architectures were included. DenseNet-121 was used to model feature reuse through dense connectivity across layers [33]. ResNet-50 was used to stabilize very deep convolutional training through residual skip connections [34]. SqueezeNet was used as a lightweight convolutional network that employed channel-wise squeeze and expand operations to reduce parameter count [35]. CapsuleNet was used to encode part–whole relationships through vector capsules and dynamic routing [36]. Xception was used as a depth-wise separable convolutional architecture that factorized spatial and channel-wise filtering [37]. These models were trained on the mushroom regions of interest to provide single-branch visual baselines. Second, two wavelength selection strategies were applied for traditional machine learning. Successive projections algorithm (SPA) was used to iteratively select a minimal, non-collinear subset of wavelengths [38]. Competitive adaptive reweighted sampling (CARS) was used to select informative wavelengths by repeatedly weighting and eliminating weak spectral variables under a regression-driven criterion [39]. Third, classical classifiers were applied to either the full spectrum or to the SPA and CARS subsets. Linear discriminant analysis (LDA) was used to project the data into a linear subspace that maximized between-class separation under a shared covariance assumption [40]. Logistic regression (LR) was used as a multinomial linear decision model optimized by cross-entropy [41]. Principal component analysis combined with linear discriminant analysis (PCA-LDA) was used to first reduce dimensionality through an unsupervised principal component transform and then apply LDA in the reduced space [42]. Partial least squares discriminant analysis (PLS-DA) was used to learn latent variables that maximized covariance between spectra and class labels under a supervised linear projection [43]. Stochastic gradient descent logistic regression (SGD-Log) was used as a linear logistic classifier trained with iterative gradient updates for efficiency on high-dimensional inputs [44]. Together, these deep learning backbones, wavelength selection strategies, and linear classifiers formed the comparison framework against which the proposed network was evaluated.

### 2.6. Model Evaluation

Model training and evaluation were conducted under a repeated, stratified hold-out protocol. For each modality combination, 10 independent trials were executed; in every trial the dataset was stratified into training/validation/test splits with 20% held out for testing and 25% of the remaining training portion used for validation. Class labels {1, 3, 5, 7, 9} were mapped to {0, 1, 2, 3, 4} and one-hot encoded for five-class softmax classification. Spectral and color–texture vectors were standardized with a scaler fit on the training split only and applied to validation/test splits. The network was optimized with Adam (initial learning rate 1 × 10^−4^), categorical cross-entropy (from logits) as the loss, and mini-batches of 16 for a maximum of 50 epochs per trial. Early stopping (patience = 12, restoring the best weights) and ReduceLROnPlateau (monitoring validation QWK, mode = max, factor = 0.5, patience = 6) were used to select models; during training, accuracy and an online quadratic weighted kappa (QWK) metric were tracked. After training, the held-out test set was evaluated to report overall accuracy, macro-averaged precision, recall, and F1-score, and a mean absolute error in days. A confusion matrix (5 classes) and wall-clock inference time were also recorded. For each experiment, the above test metrics were summarized across the 10 independent trials and are presented as the mean ± standard deviation (SD). It should be noted that because the proposed PAMIF-Net achieved a performance ceiling with zero variance across the trials, the assumption of homogeneity of variances required for standard parametric statistical tests (ANOVA) was structurally violated. Consequently, the performance comparison between models was directly evaluated based on the stability of their means and SDs rather than inferential hypothesis testing. A repeated stratified hold-out protocol was executed for 10 trials. For each trial, data stratification and model weight initialization were reset to provide a basic demonstration of training stability on this limited dataset. To strictly prevent data leakage, the dataset division was executed exclusively at the individual mushroom level prior to any patch extraction. This ensured that all spatial patches and multi-dimensional features originating from a single mushroom cap were kept entirely within either the training, validation, or test set. Consequently, the model was never evaluated on patches belonging to a mushroom it had seen during training, thereby validating the integrity of the hold-out testing. The consistent performance across all 10 independent repeated trials was used to verify the stability and reproducibility of the proposed model.

## 3. Results

### 3.1. Spectral Analysis

**Figure 4:**
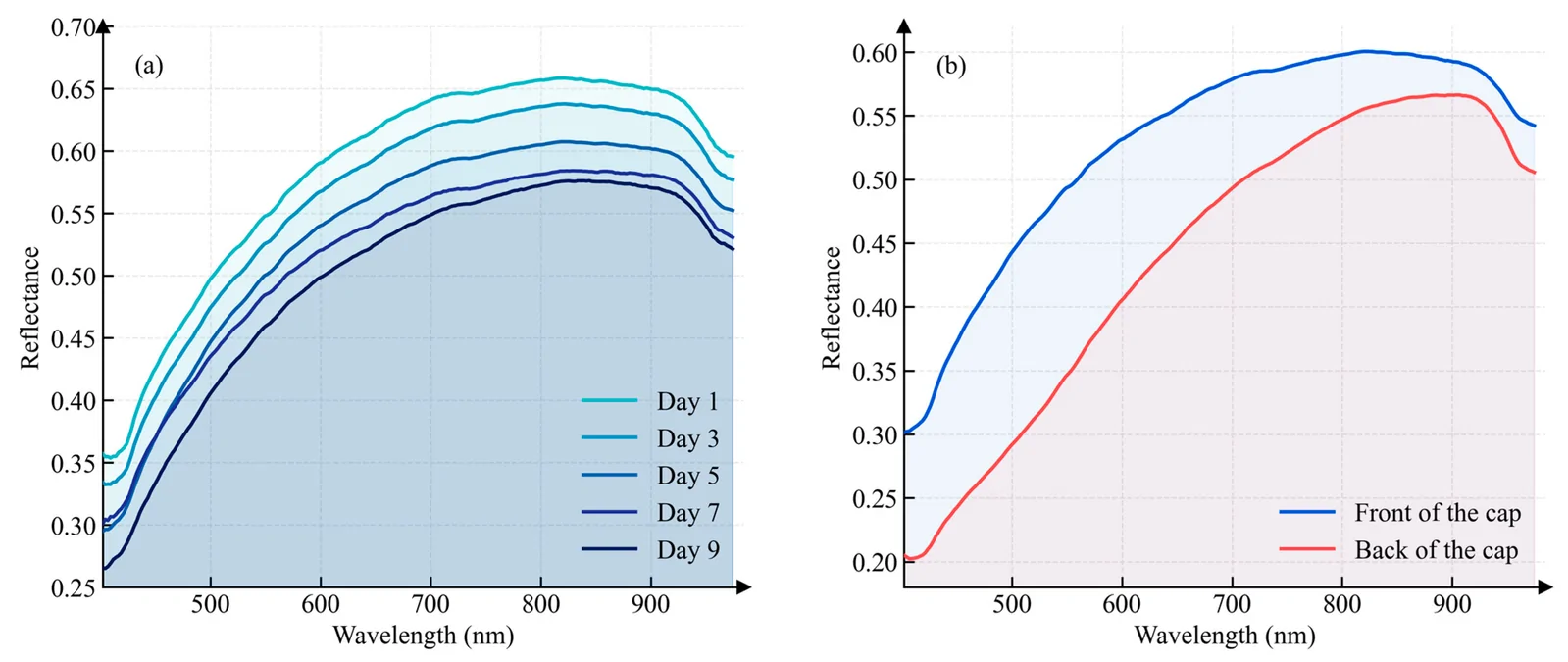
Spectral curves of the, (**a**) front of the cap on storage day 1, day 3, day 5, day 7, and day 9, (**b**) and the front versus the back (gill side) of the cap.

The reflectance curves were observed to increase monotonically from 400 to about 900–950 nm and then to decrease slightly toward 975–1000 nm, as depicted in Figure 4. The initial rise was interpreted as the combined effect of weak intrinsic absorption in the visible–NIR for white caps and strong multiple scattering from cell walls, air–tissue interfaces, and cuticle microstructures, which collectively increased albedo; as wavelength entered the longer-NIR, water-related absorption became more prominent and the net reflectance therefore declined. A weak spectral feature around ~960–975 nm was attributed to the first overtone/combination absorption of the O–H bond in water, which introduced additional attenuation and caused the slight downturn of the continuum in this region [45]. With increasing storage time, the spectrum shifted downward. It must be emphasized that the proposed model classifies freshness based primarily on these captured optical and behavioral manifestations rather than directly quantifying the underlying chemical concentrations. However, based on established spectroscopic principles, this broadband downward shift is widely attributed in the literature to progressive enzymatic browning and the synthesis of melanin-like pigments, which increase broad absorption across the visible range [23,46,47]. Microstructural compaction and surface roughening further reduced backscattering efficiency. In addition, water redistribution or exudation enhanced NIR attenuation around the O–H bands [22,28]. Consequently, reflectance decreased across most wavelengths from day 1 to day 9. Anatomically, the front exhibited much higher reflectance than the back: the front side possessed a smoother, more coherent, and less pigmented cuticle with lower effective pathlengths and fewer occlusions, whereas the gill side contained densely packed lamellae with higher moisture content and complex micro-geometry, both of which increased internal absorption and shadowing; consequently, the back appeared systematically darker than the front over 400–1000 nm.

### 3.2. Unsupervised Methods Analysis

**Figure 5:**
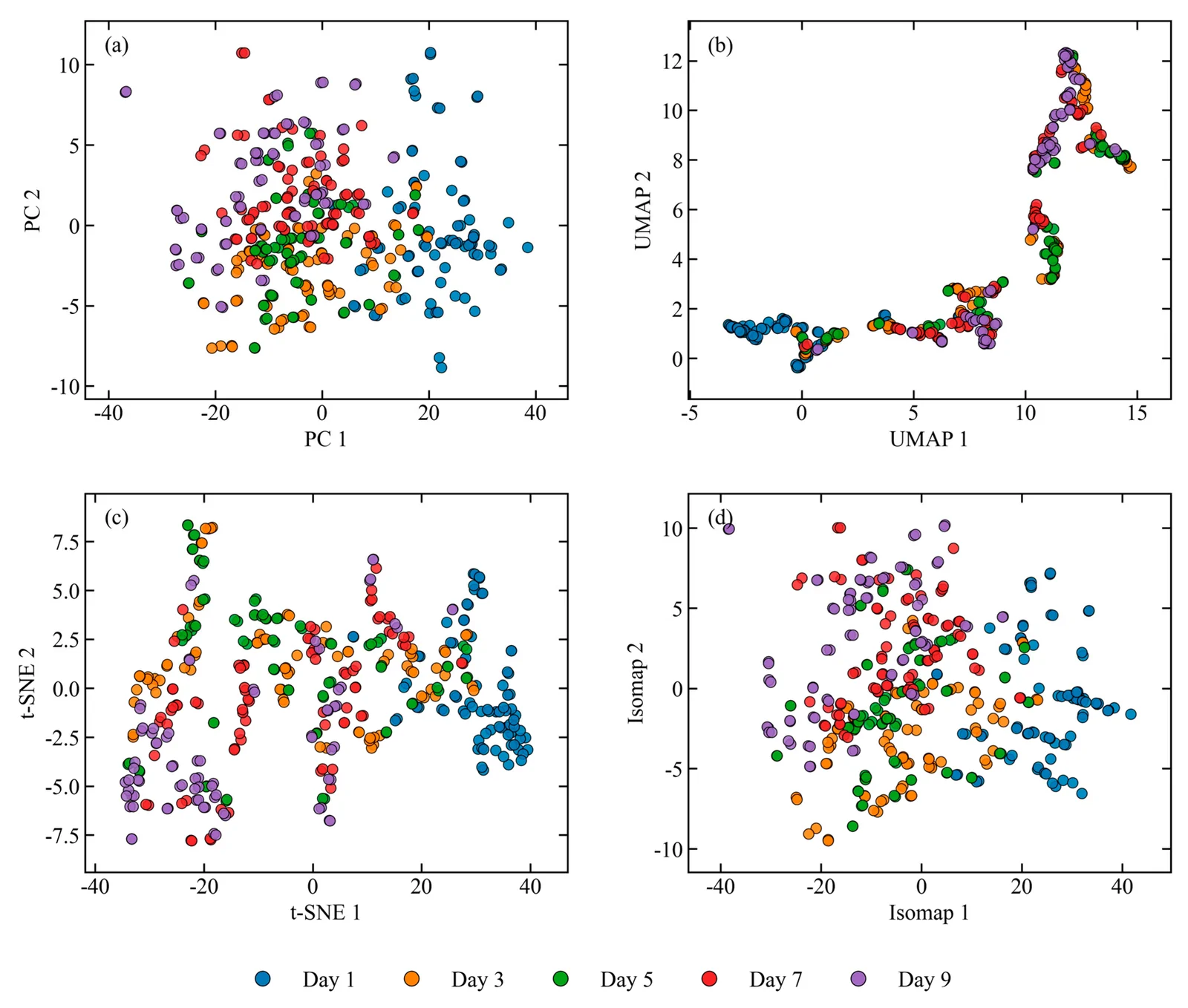
Visualization of unsupervised methods: (**a**) PCA; (**b**) UMAP; (**c**) t-SNE; (**d**) ISOMAP.

Four unsupervised dimensionality-reduction techniques were employed to explore the intrinsic structure of the hyperspectral data collected from mushrooms stored for day 1, day 3, day 5, day 7, and day 9, including PCA, UMAP, t-SNE, and ISOMAP. Overall, the visualizations revealed a general progression from early to late storage. However, the 5 storage periods were not distinctly separated, as shown in Figure 5. In the PCA projection, the majority of spectral variance was explained by a single continuous component associated with a gradual broadband reflectance decrease during storage, resulting in partially overlapping distributions between adjacent days rather than clearly defined clusters. Similarly, UMAP and ISOMAP mapped the samples onto a smooth manifold with considerable overlap among days 3–7, suggesting that the underlying spectral evolution followed a continuous aging trajectory rather than discrete categorical transitions. The t-SNE embedding yielded compact local clusters but intermingled neighboring storage periods, reflecting its focus on preserving local neighborhood densities rather than global chronological order.

The limited separability of storage days was mainly attributed to several intrinsic factors. First, the physicochemical changes during storage occurred gradually, producing subtle spectral shifts that were comparable in magnitude to within-day biological variability. Second, individual differences in cap microstructure, surface moisture, and orientation introduced additional variance that masked the between-day signal. Third, unsupervised algorithms emphasize variance preservation or manifold topology rather than discriminative feature learning, and thus could not maximize inter-class separability among the ordinal groups. Finally, local-structure-preserving methods such as UMAP and t-SNE tended to fragment the continuous time evolution into multiple density islands, further weakening the global trend. The high degree of overlap observed in these manifold learning methods occurs because they optimize purely for variance preservation or local topology without label guidance, thus accurately reflecting the smooth, continuous nature of biological aging as a continuum. In contrast, nonlinear supervised deep learning classifiers (such as PAMIF-Net) successfully outperform these methods by utilizing label-driven loss functions to actively warp the feature space. By leveraging spatial–spectral embeddings and gated multimodal fusion, the supervised model identifies highly discriminative, non-obvious hyperplanes (such as specific localized texture-spectra interactions) to forcefully separate continuous boundary cases into discrete classes. Consequently, although unsupervised visualization effectively confirmed the existence of a coherent aging trajectory and overall data consistency, it was inadequate for precise differentiation of the chronological storage duration, further highlighting the need for supervised models to track these time-dependent optical changes.

### 3.3. Model Performance of PAMIF-Net

The performance of PAMIF-Net was quantified under multiple modality configurations, and several consistent patterns were observed, as displayed in Table 1. When using a single branch, the spatial–spectral CNN tokenization achieved nearly perfect recognition performance (accuracy 100 ± 2%, MAE 0.02 ± 0.06 d) with the shortest runtime (9.71 s). In contrast, hand-crafted meso-scale descriptors performed poorly, with PATCH and MGAF reaching only 29 ± 10% and 49 ± 5% accuracy, respectively. These results suggested that local mean spectra and within-patch interaction features lacked sufficient discriminative capability in the absence of hierarchical feature learning. Although the global tabular cues carried more information, as indicated by the SPEC and COLOR branches reaching 83 ± 5% and 85 ± 5% accuracy, respectively, they still underperformed compared with the CNN stream. When modalities were fused, the role of patch-aligned complementarity became evident. Adding PATCH to CNN, or CNN plus MGAF jointly, yielded highly consistent recognition (100 ± 0% accuracy, 0.00 ± 0.00 d MAE) across the evaluated test splits. We rigorously re-verified the testing protocol to rule out spatial data leakage, confirming that multi-dimensional features from the same mushroom were strictly isolated from the test set. While these optimal metrics demonstrate that the multimodal fusion successfully resolved the class boundaries within the scope of this highly controlled dataset, they represent a dataset-specific performance ceiling. Real-world applications involving unconstrained environmental fluctuations may exhibit greater variance; nevertheless, the results prove that meso-scale heterogeneity and inter-component relations are highly informative when guided by learned spatial–spectral features. Other CNN-centric fusions (CNN + SPEC, CNN + COLOR, CNN + SPEC + COLOR) maintained ≥98.88% accuracy with MAE ≤ 0.04 d, confirming that global spectra and color acted as stable conditioners that refined decision margins rather than redefining them. In contrast, fusions without CNN remained mediocre and unstable (e.g., PATCH + MGAF 43.12%, PATCH + SPEC + COLOR 81.12%), underscoring that PAMIF-Net’s gains were driven primarily by the tokenized CNN branch with gated, patch-registered integration of complementary cues. Runtime increased monotonically with added branches (e.g., CNN + PATCH + MGAF, 25.45 s; ALL(light), 33.17 s), revealing a clear accuracy–efficiency trade-off in which CNN-only or CNN + SPEC offered strong baselines for time-constrained use, while CNN + PATCH (or CNN + PATCH + MGAF) delivered maximal accuracy for applications prioritizing reliability. Collectively, these results showed that freshness recognition benefited most when subtle, distributed spatial–spectral motifs were learned and fused. It is important to note that the baseline models and isolated branches frequently failed in worst-case scenarios, specifically, misclassifying boundary cases such as Day 3 mushrooms experiencing accelerated moisture loss as Day 5, or robust Day 7 samples as Day 5. The high accuracy achieved by PAMIF-Net (reaching 100% within the scope of this specific dataset) is not an indication of absolute perfection in all real-world conditions, but rather demonstrates that the network’s gated fusion successfully resolved these specific boundary ambiguities by cross-referencing local spectral interactions (MGAF) with global texture, preventing the model from being misled by a single atypical feature. It is critical to note that while PAMIF-Net achieved an optimal 100% accuracy and 0.00 MAE on the held-out test sets, this exceptional metric should be interpreted as a dataset-specific performance ceiling rather than an infallible universal capability. Post-harvest biological samples possess inherent physiological variability. The optimal separation observed here was significantly facilitated by the strictly controlled environmental conditions (constant 4 °C and humidity) and the discrete 48 h sampling intervals, which minimized overlap. In real-world cold chains with fluctuating conditions, biological variability would inevitably reintroduce class boundary overlap, leading to a natural decrease in accuracy.

### 3.4. Ablation Test

In the ablation test, a patch-aligned two-branch baseline was constructed by fusing the CNN token stream with the PATCH stream. Single-factor variants were then generated by removing the squeeze–excitation (SE) module in one or both branches, replacing the attention-weighted fusion with concatenation or mean fusion, substituting batch normalization with layer normalization, and modifying the network depth. Each variant was trained and evaluated through ten independent stratified trials using fixed train–validation–test splits. The performance metrics on the test set were calculated and summarized across all trials, as depicted in Table 2. The base model achieved 100.00% accuracy with 0.00 d MAE and 12.29 s runtime, which established the performance ceiling in this setting. Removing all SE units reduced the accuracy to 99.50% and increased the MAE to 0.02 d. When the SE module was removed only from the CNN branch, the accuracy slightly improved to 99.88% with an MAE of 0.01 d. In contrast, removing SE only from the PATCH branch maintained perfect performance with 100.00% accuracy and 0.00 d MAE. These results indicated that channel recalibration contributed to feature enhancement, although its effect was modest and dependent on the specific branch. Replacing the attention-weighted fusion with concatenation resulted in 99.88% accuracy and an MAE of 0.01 d. Using mean fusion achieved the same accuracy and MAE values. When a linear attention mechanism was applied, the model reached 100.00% accuracy with an MAE of 0.00 d. These results suggested that the fusion head was highly robust and that the nonlinearity in the attention gate was not essential under the current data distribution. Switching batch normalization to layer normalization preserved 100.00% accuracy and 0.00 d MAE, but the runtime increased to 21.81 s, which implied a clear efficiency penalty without accuracy gains. Making the network shallow maintained perfect accuracy at 100.00% and achieved the fastest runtime of 9.32 s. In contrast, increasing the network depth reduced the accuracy to 99.62% and extended the runtime to 24.73 s. This result indicated that excessive parameterization was unnecessary and could even have a negative impact on model performance. Taken together, these results showed that the CNN + PATCH backbone with batch normalization, SE modules, and light attention fusion was sufficient to capture the essential spatial–spectral features. The SE and attention mechanisms provided additional stability but did not lead to substantial performance gains. Moreover, the normalization strategy and network depth primarily influenced computational efficiency rather than recognition accuracy in this task.

### 3.5. Comparative Test of Classic Deep Learning Models

**Figure 6:**
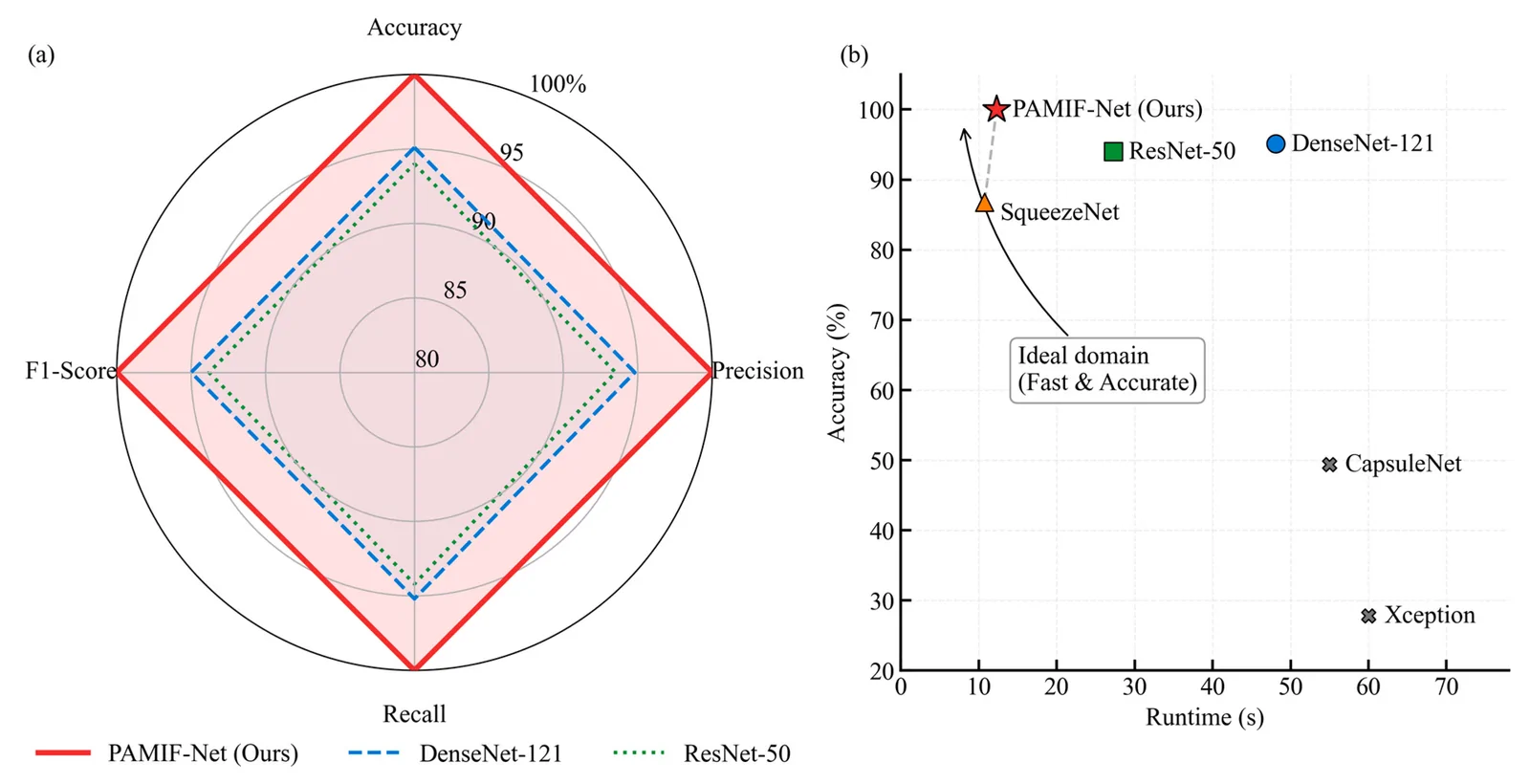
Performance comparison of PAMIF-Net and classic deep learning models: (**a**) multidimensional evaluation metrics; (**b**) accuracy versus runtime trade-off.

In the comparative test, PAMIF-Net was evaluated against several widely used deep learning models, including DenseNet-121, ResNet-50, SqueezeNet, CapsuleNet, and Xception, as shown in Table 3. Identical input representations, preprocessing pipelines, and feature engineering procedures were adopted for all compared deep learning models in this comparative test, to ensure the fairness and objectivity of the performance comparison. As illustrated in the multidimensional radar chart (Figure 6a), PAMIF-Net yielded higher performance metrics on this dataset, achieving 100% across the evaluated metrics (accuracy, precision, recall, and F1-score) for the given test sets. Notably, the absolute consistency (zero variance) of PAMIF-Net’s performance precluded the use of traditional parametric statistical comparisons; however, its absolute stability and superior mean metrics distinctly separated it from classical backbones, such as DenseNet-121 (95.1 ± 6.7%) and ResNet-50 (94.0 ± 12.0%), which exhibited significant fluctuation. CapsuleNet and Xception performed poorly, with 49.4% and 27.8% accuracy, respectively. It is worth noting that the suboptimal performance of CapsuleNet and Xception does not imply inherent flaws within these models; rather, their specific architectural frameworks were fundamentally ill-suited for capturing the unique high-dimensional and spectral-spatial characteristics of the dataset used in this study.

To further evaluate the practical deployability of the algorithms, a Pareto trade-off landscape was constructed to map the relationship between model accuracy and computational runtime (Figure 6b). Traditionally, a trade-off exists between operational speed and prediction accuracy, as evidenced by SqueezeNet (lower latency but lower accuracy) and DenseNet-121 (higher accuracy but computationally intensive). On this dataset, PAMIF-Net mitigated this trade-off. As depicted in Figure 6b, the runtime of PAMIF-Net was 12.29 ± 1.43 s, which was markedly lower than DenseNet-121 at 48.08 s and ResNet-50 at 27.26 s, and was comparable to SqueezeNet at 10.74 s. These results indicated that PAMIF-Net delivered both higher accuracy and better efficiency than most conventional architectures.

Furthermore, the detailed learning dynamics, presented as supplementary learning curves (Figure S1), confirmed that PAMIF-Net reached high training accuracy within the first few epochs and subsequently maintained exceptional stability without overfitting, whereas other networks required significantly more epochs to converge or suffered from persistent oscillation. The observed advantage was attributed to several design factors. First, PAMIF-Net operated on a patch-aligned, mushroom-level representation that preserved localized freshness cues rather than relying solely on global appearance. Second, spatial–spectral tokens from the CNN branch were explicitly fused with meso-scale descriptors, which enhanced discrimination of subtle and spatially heterogeneous aging patterns on the cap surface. Third, the lightweight gated fusion suppressed redundant information and emphasized stable features related to browning, moisture loss, and texture changes. Taken together, PAMIF-Net provided fast convergence, high stability, and near-perfect recognition of storage day, while generic CNN backbones and capsule-based models were less able to extract these subtle, storage-related signatures from hyperspectral inputs.

### 3.6. Comparative Test of Machine Learning Models

In the comparative test of machine learning models, three types of input features were evaluated. The full-wavelength (FW) reflectance contained all 226 spectral bands. The SPA and the CARS were then used to perform wavelength selection. SPA extracted 16 characteristic wavelengths and CARS extracted 32 characteristic wavelengths, and the distributions of these selected wavelengths were shown in Figure S2. These features were classified using traditional algorithms, including LDA, LR, PCA-LDA, PLS-DA, and SGD-Log. The results in Table 4 showed that the use of all 226 bands with LR achieved the best overall performance among the traditional methods, with 98.00% accuracy, 98.00% precision, 98.00% recall, and 98.00% F1-score. When dimensionality was reduced by wavelength selection, performance declined in most cases. With SPA, which used only 16 wavelengths, the best model reached 94.00% accuracy with 94.14% precision and 94.00% recall. With CARS, which used 32 wavelengths, LR reached 97.00% accuracy and 96.99% precision. Models based on PCA-LDA and PLS-DA showed much lower performance under all feature configurations. These findings indicated that linear decision boundaries applied to the full spectrum still captured most of the class structure, and that aggressive wavelength reduction introduced an accuracy loss that was only partially recoverable. When compared with PAMIF-Net, which achieved 100.00% accuracy with 100.00% precision, recall, and F1-score, the gap was clear. PAMIF-Net outperformed all classical pipelines even though some of those pipelines used the complete spectral information. This advantage was attributed to two factors. First, PAMIF-Net did not rely solely on global spectra. Instead, it jointly modeled localized spectral states, spatial texture, and patch-level heterogeneity on the mushroom cap surface, which classical models could not represent. Second, PAMIF-Net fused these heterogeneous cues through a learned multimodal attention mechanism and produced spatially aware tokens that were aligned with physical regions on the cap. As a result, PAMIF-Net exploited subtle, spatially non-uniform aging patterns such as localized browning and moisture loss, whereas traditional machine learning models treated each mushroom as a single averaged spectrum and therefore missed these fine-grained deterioration signatures. Furthermore, the superior performance of PAMIF-Net aligns with and expands upon findings from earlier postharvest studies. Previous research has often targeted isolated quality attributes, such as browning detection utilizing visible spectrum colorimetry [23,46] or moisture loss estimation focusing on NIR water absorption bands around 970 nm [22]. While those studies successfully correlated specific spectral features with individual deterioration parameters, they typically relied on averaged spectra that mask structural degradation. Postharvest deterioration of *A. bisporus*, however, is a concurrent physiological process involving simultaneous enzymatic browning, tissue water exudation, and texture softening [11]. By dynamically fusing global spectral trends (capturing overall moisture and chemical shifts) with meso-scale spatial textures (capturing localized browning and morphological collapse), PAMIF-Net effectively models the synergistic effects of these pathways. This multimodal capacity explains its significantly higher classification stability for overall shelf-life prediction compared to traditional single-domain analyses reported in earlier literature.

## 4. Conclusions

The hyperspectral signatures of button mushrooms during refrigerated storage were characterized, and progressive browning, moisture loss, and structural degradation were reflected as a gradual decline in reflectance and as increasing spatial heterogeneity across the cap surface. It was shown that unsupervised methods could reveal an overall aging trajectory but could not clearly separate individual storage days, which indicated that mushroom deterioration proceeded as a continuous process rather than as discrete states. To overcome this limitation, a multi-dimensional feature construction strategy was applied to each hyperspectral region of interest, and complementary descriptors of global spectra, surface color and texture, local patch-level spectral states, intra-patch spectral interactions, and learned spatial–spectral tokens were extracted and fused. This fusion was implemented through a patch-aligned multimodal interaction fusion network, PAMIF-Net, which resolved the boundary misclassifications prevalent in simpler models and achieved high recognition accuracy of five storage times on the evaluated dataset, consistently outperforming both standard convolutional backbones and classical machine learning models. The network was further shown to maintain high accuracy at practical inference cost, which suggested that rapid, objective, and non-destructive estimation of remaining shelf-life could be achieved at the level of single caps. In conclusion, these findings indicated that postharvest quality evaluation of mushrooms could be advanced from manual appearance-based grading to automated freshness verification using hyperspectral imaging and structured spectral–spatial fusion. This advancement serves as a foundational proof-of-concept, demonstrating that postharvest classification can benefit from such integrated non-destructive technologies. While further research is required to adapt this principle to real-world, fluctuating supply chain environments, it provides a promising methodological framework for future non-destructive quality evaluation.

Despite these promising results, the current study is constrained by evaluating performance solely based on chronological storage duration rather than direct biochemical degradation. Because biological variability causes individual mushrooms to age at different rates, storage time serves only as an indirect proxy for freshness. A critical limitation of this framework is the absence of independent physicochemical reference parameters. Future studies must incorporate parallel destructive assays, such as precise measurements of weight loss (%), tissue firmness (N), browning index, and polyphenol oxidase (PPO) activity, to robustly correlate the learned spatial–spectral signatures with objective biochemical degradation metrics, thereby fully validating the practical relevance of the model for food science. Furthermore, because the hyperspectral data were acquired under uniform laboratory illumination, the model’s robustness must be further validated against real-world industrial constraints. Although the inclusion of Local Binary Patterns (LBP) and CIE-Lab color space within PAMIF-Net theoretically provides resilience against moderate lighting drifts, and patch-alignment helps mitigate shadows caused by spherical cap geometry, unconstrained cold-chain environments pose more severe lighting heterogeneities. Additionally, while the ~12s inference time per image is highly competitive among deep learning frameworks, achieving real-time, continuous processing for high-throughput logistics will require further optimization through model quantization and edge-computing deployment.

## Figures and Tables

**Table 1:** Model performance of PAMIF-Net.

Model	Accuracy (%)	Precision (%)	Recall (%)	F1-Score (%)	MAE (d)	Runtime (s)
CNN only	99.50 ± 1.58	99.60 ± 1.26	99.50 ± 1.58	99.49 ± 1.61	0.02 ± 0.06	9.71 ± 2.23
PATCH only	28.75 ± 9.61	14.25 ± 9.85	28.75 ± 9.61	16.25 ± 9.32	2.87 ± 0.81	9.84 ± 0.34
MGAF only	48.88 ± 5.35	41.55 ± 10.21	48.88 ± 5.35	41.77 ± 7.12	1.24 ± 0.17	26.10 ± 7.80
SPEC only	82.88 ± 5.14	83.39 ± 5.32	82.88 ± 5.14	82.47 ± 5.47	0.47 ± 0.15	10.83 ± 2.18
COLOR only	84.50 ± 5.34	85.08 ± 5.19	84.50 ± 5.34	84.26 ± 5.54	0.72 ± 0.28	10.76 ± 1.90
CNN + PATCH	100.00 ± 0.00	100.00 ± 0.00	100.00 ± 0.00	100.00 ± 0.00	0.00 ± 0.00	12.29 ± 1.43
CNN + MGAF	99.38 ± 1.35	99.42 ± 1.25	99.38 ± 1.35	99.37 ± 1.35	0.02 ± 0.04	18.68 ± 1.88
CNN + SPEC	99.12 ± 1.32	99.18 ± 1.23	99.12 ± 1.32	99.11 ± 1.35	0.03 ± 0.04	10.90 ± 0.83
CNN + COLOR	98.88 ± 1.24	98.95 ± 1.14	98.88 ± 1.24	98.86 ± 1.27	0.04 ± 0.04	10.16 ± 0.60
PATCH + MGAF	43.12 ± 16.39	32.11 ± 24.34	43.12 ± 16.39	32.12 ± 19.51	1.61 ± 0.67	29.35 ± 13.83
SPEC + COLOR	87.62 ± 3.03	88.36 ± 2.91	87.62 ± 3.03	87.61 ± 3.08	0.45 ± 0.09	14.47 ± 2.17
CNN + SPEC + COLOR	99.25 ± 1.21	99.33 ± 1.04	99.25 ± 1.21	99.25 ± 1.22	0.02 ± 0.03	12.05 ± 0.81
CNN + PATCH + MGAF	100.00 ± 0.00	100.00 ± 0.00	100.00 ± 0.00	100.00 ± 0.00	0.00 ± 0.00	25.45 ± 2.51
PATCH + SPEC + COLOR	81.12 ± 15.21	82.10 ± 14.37	81.12 ± 15.21	80.74 ± 15.56	0.65 ± 0.57	23.84 ± 9.81
CNN + PATCH + MGAF + SPEC	98.88 ± 1.50	98.92 ± 1.45	98.88 ± 1.50	98.86 ± 1.53	0.04 ± 0.06	31.98 ± 3.01
CNN + PATCH + MGAF + COLOR	98.50 ± 2.02	98.60 ± 1.86	98.50 ± 2.02	98.51 ± 2.00	0.05 ± 0.07	30.71 ± 2.97
ALL(light)	98.38 ± 2.43	98.49 ± 2.23	98.38 ± 2.43	98.38 ± 2.42	0.06 ± 0.09	33.17 ± 3.27

**Table 2:** Model performance of PAMIF-Net in ablation test.

No.	Model	Accuracy (%)	Precision (%)	Recall (%)	F1-Score (%)	QWK	MAE (d)	Runtime (s)
1	BASE	100.00 ± 0.00	100.00 ± 0.00	100.00 ± 0.00	100.00 ± 0.00	100.00 ± 0.00	0.00 ± 0.00	12.29 ± 1.43
2	NoSE	99.50 ± 1.05	99.54 ± 0.97	99.50 ± 1.05	99.50 ± 1.06	99.53 ± 1.28	0.02 ± 0.04	10.92 ± 0.73
3	NoSE_CNN	99.88 ± 0.40	99.88 ± 0.37	99.88 ± 0.40	99.87 ± 0.40	99.72 ± 0.90	0.01 ± 0.02	11.52 ± 0.85
4	NoSE_PATCH	100.00 ± 0.00	100.00 ± 0.00	100.00 ± 0.00	100.00 ± 0.00	100.00 ± 0.00	0.00 ± 0.00	12.20 ± 1.07
5	FUSE_Concat	99.88 ± 0.40	99.88 ± 0.37	99.88 ± 0.40	99.87 ± 0.40	99.88 ± 0.39	0.01 ± 0.02	11.98 ± 0.74
6	FUSE_Mean	99.88 ± 0.40	99.88 ± 0.37	99.88 ± 0.40	99.87 ± 0.40	99.72 ± 0.88	0.01 ± 0.02	13.07 ± 1.14
7	FUSE_AttnLinear	100.00 ± 0.00	100.00 ± 0.00	100.00 ± 0.00	100.00 ± 0.00	100.00 ± 0.00	0.00 ± 0.00	12.95 ± 0.92
8	Norm_LN	100.00 ± 0.00	100.00 ± 0.00	100.00 ± 0.00	100.00 ± 0.00	100.00 ± 0.00	0.00 ± 0.00	21.81 ± 2.34
9	Shallow	100.00 ± 0.00	100.00 ± 0.00	100.00 ± 0.00	100.00 ± 0.00	100.00 ± 0.00	0.00 ± 0.00	9.32 ± 1.35
10	Deep	99.62 ± 0.60	99.65 ± 0.57	99.62 ± 0.60	99.62 ± 0.60	98.94 ± 1.81	0.03 ± 0.04	24.73 ± 1.65

**Table 3:** Performance of other deep learning models.

Model	Accuracy (%)	Precision (%)	Recall (%)	F1-Score (%)	Runtime (s)
PAMIF-Net	100.00 ± 0.00	100.00 ± 0.00	100.00 ± 0.00	100.00 ± 0.00	12.29 ± 1.43
DenseNet−121	95.1 ± 6.69	95.19 ± 6.59	95.1 ± 6.69	95.14 ± 6.64	48.08 ± 2.47
ResNet−50	94.0 ± 12.03	94.03 ± 11.89	93.6 ± 12.67	93.81 ± 12.29	27.26 ± 1.26
SqueezeNet	86.8 ± 11.95	89.4 ± 11.24	81.9 ± 18.19	84.73 ± 14.11	10.74 ± 1.1
CapsuleNet	49.4 ± 4.43	81.74 ± 10.16	17.1 ± 5.04	27.91 ± 6.91	5.41 ± 0.85
Xception	27.8 ± 14.31	35.35 ± 26.49	20.8 ± 6.11	23.48 ± 6.8	29.71 ± 1.39

**Table 4:** Performance of traditional machine learning models.

Data	Model	Accuracy (%)	Precision (%)	Recall (%)	F1-Score (%)
FW	LDA	95.00	95.14	95.00	94.90
FW	LR	98.00	98.00	98.00	98.00
FW	PCA-LDA	58.00	56.13	58.00	56.26
FW	PLS-DA	48.00	28.38	48.00	35.57
FW	SGD-Log	83.00	84.59	83.00	82.91
SPA	LDA	85.00	85.64	85.00	84.96
SPA	LR	94.00	94.14	94.00	94.05
SPA	PCA-LDA	74.00	73.42	74.00	72.96
SPA	PLS-DA	51.00	43.08	51.00	42.50
SPA	SGD-Log	60.00	67.25	60.00	59.01
CARS	LDA	84.00	85.00	84.00	84.23
CARS	LR	97.00	96.99	97.00	96.97
CARS	PCA-LDA	60.00	58.85	60.00	58.97
CARS	PLS-DA	52.00	49.74	52.00	43.35
CARS	SGD-Log	75.00	75.14	75.00	73.37

## Data Availability

The original contributions presented in this study are included in the article/Supplementary Materials. Further inquiries can be directed to the corresponding authors.

## Supplementary Materials

The following supporting information can be downloaded at https://www.mdpi.com/article/10.3390/foods15183224/s1: Figure S1: Learning curves of PAMIF-Net and deep learning models: (a) training accuracy curves; (b) validation accuracy curves. Figure S2: Characteristic wavelength distribution: (a) SPA; (b) CARS.
