# Supplementary material for: A Patch-Aligned Multimodal Deep Learning Framework for Non-Destructive Freshness Monitoring of Postharvest Mushrooms Using Hyperspectral Imaging
Source: Foods. 2026 Sep 11;15(18):3224. doi: 10.3390/foods15183224 (PMC13606851; doi:10.3390/foods15183224)
Supplement: Supplementary file 1 [file foods-15-03224-s001.zip › foods-4523573-supplementary.pdf]

A Patch-Aligned Multimodal Deep Learning Framework for Non-Destructive Freshness Monitoring of Postharvest Mushrooms Using Hyperspectral Imaging

Zhen Guo [1,*], Yaru Wang [1], Lele Cao [1], Fernando A. Auat-Cheein [2,3], Xingfeng Guo [1,*]

[1] Shandong Key Laboratory of Applied Technology for Protein and Peptide Drugs, School of Pharmaceutical Sciences and Food Engineering, Liaocheng University, Liaocheng 252000, China.

[2] Department of Electronic Engineering, Universidad Técnica Federico Santa María, Av. España 1680, Valparaiso 2390123, Chile.

[3] Department of Agricultural Engineering, Harper Adams University, Newport, Shropshire TF10 8NB, UK.

[*] Corresponding authors E-mail: guozhen@lcu.edu.cn (Zhen Guo) and guoxingfeng@lcu.edu.cn (Xingfeng Guo).

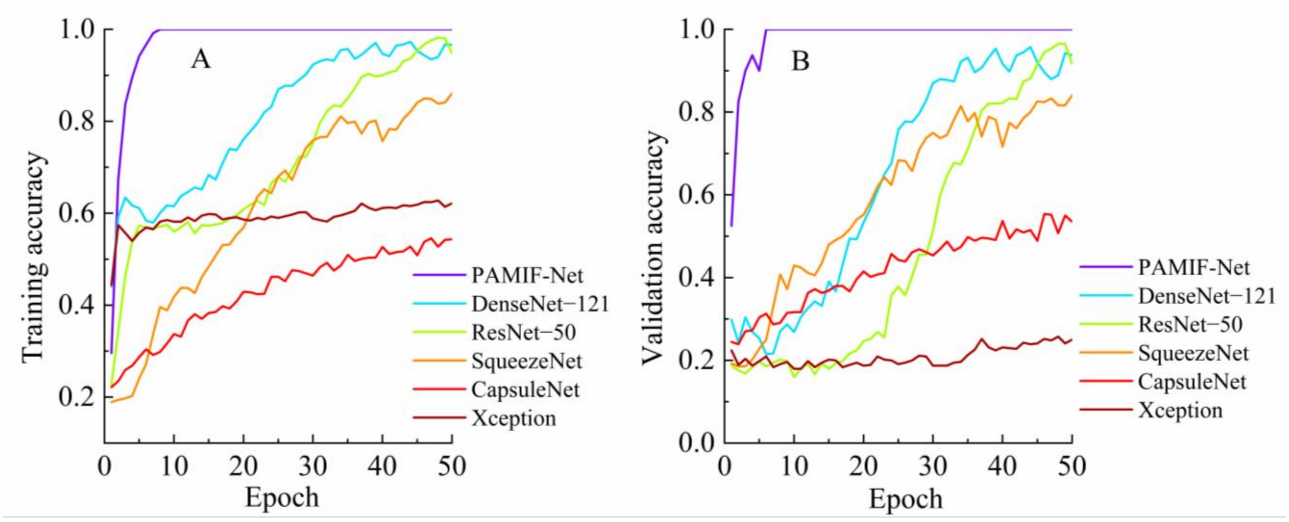

Figure S1 Learning curves of PAMIF-Net and deep learning models, (a) training accuracy curves,

(b) validation accuracy curves

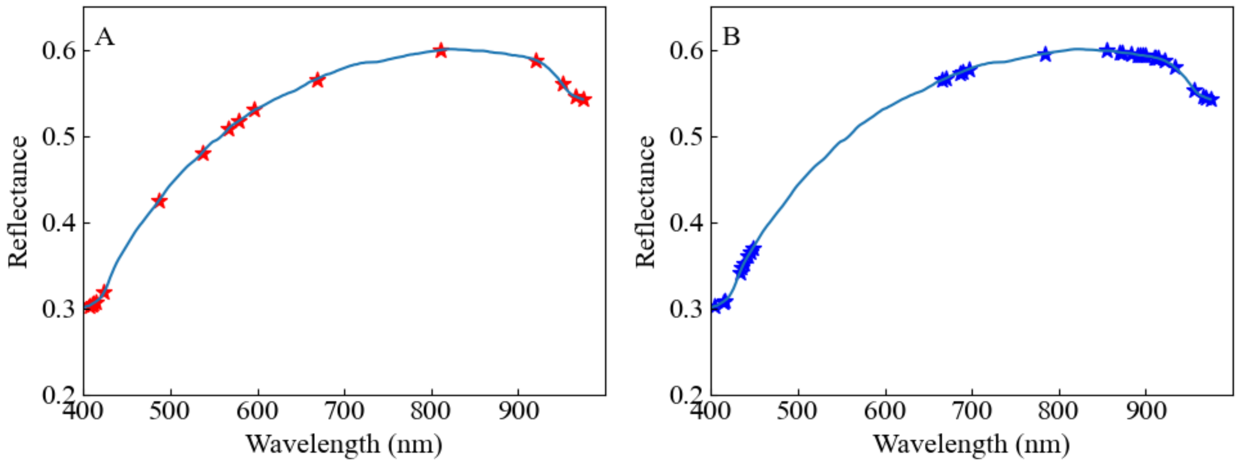

Figure S2 Characteristic wavelength distribution, (a) SPA, (b) CARS
